# Integrated transcriptomic and metabolomic analysis of provenances and organ-specific responses to drought and rehydration in *Pinus tabuliformis*

**DOI:** 10.3389/fpls.2026.1778743

**Published:** 2026-03-12

**Authors:** Shuwei Zhang, Xu Wang, Siyu Liu, Zhiyu Zhang, Yanan Yu, Tong Wu, Yongsheng Chen, Yuhan Chen, Xiangyu Meng, Wei Li, Wenhao Bo

**Affiliations:** State Key Laboratory of Tree Genetics and Breeding, National Engineering Research Center of Tree Breeding and Ecological Restoration, Key Laboratory of Genetics and Breeding in Forest Trees and Ornamental Plants, Ministry of Education, Center for Computational Biology, College of Biological Sciences and Technology, Beijing Forestry University, Beijing, China

**Keywords:** drought stress, metabolomics, organ-specific response, *Pinus tabuliformis*, provenances, transcriptomics

## Abstract

**Introduction:**

Drought stress severely limits the survival and growth of *Pinus tabuliformis* seedlings during afforestation. However, the molecular mechanisms coordinating organ-specific responses across diverse geographical provenances remain inadequately characterized.

**Methods:**

This study investigated the dynamic drought responses of two provenances of *P. tabuliformis*, originating from semi-arid (HLH) and semi-humid (SL) regions, through a time-course drought and rewatering experiment. Integrated transcriptomic and metabolomic analyses of roots and leaves revealed a clear functional specialization between these organs.

**Results:**

Roots primarily initiated signal transduction and osmotic adjustment, whereas needles were predominantly engaged in minimizing water loss through transpiration. Notably, the HLH provenance demonstrated a rapid-response plasticity strategy, while the SL provenance exhibited a highly dynamic defensive strategy. Several key candidate genes, including Pt2G21940, Pt1G23820, and Pt5G10920, were identified. Furthermore, gene-metabolite correlation networks underpinning the dynamics of proline, jasmonic acid, and flavonoid metabolism were delineated.

**Discussion:**

These findings provide a molecular foundation for deciphering the drought resistance mechanisms in *P. tabuliformis* and offer valuable genetic targets for breeding drought-tolerant conifers.

## 1 Introduction

Anthropogenic warming has significantly accelerated global drought severity, with atmospheric evaporative demand increasing drought intensity by approximately 40% (Gebrechorkos et al., 2025). These compound extremes, notably the 2022 and 2023 heatwave and drought events, triggered widespread forest canopy dieback and mortality pulses, exemplified by recent events in North China (Chen et al., 2025). Conifers inhabiting cold or arid environments with limited resources face severe growth decline arising from functional compromises essential for tolerating multiple stressors (McCulloh et al., 2023). Furthermore, the increasing duration and intensity of droughts render gymnosperm forests in dry regions highly susceptible to hydraulic failure and ecosystem collapse (Gazol et al., 2025).

Following exposure to drought stress, plants undergo various morphological and structural modifications to enhance water-use efficiency (WUE) and overall survival. The root system serves as the primary organ for perceiving soil moisture fluctuations. Under water deficit, roots promote vertical growth to access moisture in deeper soil layers, while the aerial parts reduce water requirements by modulating stomatal closure and decreasing total leaf area.

Plants utilize a suite of biochemical, physiological, and molecular responses across whole-plant, organ, tissue, and cellular levels to mitigate drought stress (Nour et al., 2024; Dao et al., 2025). At the physiological level, drought significantly impairs chlorophyll synthesis, nutrient metabolism, and ion homeostasis, thereby reducing photosynthetic rates (Haghpanah et al., 2024). Drought also triggers the production of reactive oxygen species (ROS) as early signaling molecules. However, the excessive accumulation of ROS induces oxidative stress, leading to ion imbalance, protein denaturation, and oxidative damage to nucleic acids (Suzuki et al., 2012; Wang et al., 2019). Enzymatic antioxidants, including ascorbate peroxidase (APX), superoxide dismutase (SOD), catalase (CAT), and glutathione reductase (GR), are essential for maintaining ROS homeostasis and play a critical role in alleviating drought-induced damage (Mishra et al., 2023).

Drought tolerance is a complex quantitative trait regulated by coordinated gene networks involved in stress perception, signal transduction, and amplification (Liu et al., 2025). Regulatory mechanisms are generally categorized into abscisic acid (ABA)-dependent and ABA-independent pathways. The ABA signaling pathway acts as a central regulator, utilizing a cascade involving PYR/PYL/RCAR receptors, PP2Cs, and SnRK2s to precisely modulate stomatal movement, gene expression, and root architecture under water deficit (Aslam et al., 2022). Concurrently, calcium (Ca^2+^) signaling and mitogen-activated protein kinase (MAPK) cascades serve as pivotal systems for translating environmental stimuli into cellular responses (Yang et al., 2021). These mediators activate key transcription factors, such as *MYB* and *NAC*, to regulate the biosynthesis of secondary metabolites, including terpenoids and flavonoids (Nakano et al., 2015). Furthermore, this transcriptional regulation operates in coordination with hormonal signaling, such as ABA and jasmonic acid (JA), to synchronize stomatal aperture and antioxidant activities for ROS scavenging (Jardim-Messeder et al., 2023; Liu et al., 2018; Li et al., 2024). These hierarchical mechanisms constitute a systemic regulatory network where stress induces dynamic changes in transcriptomic and metabolomic profiles. Consequently, the integrated analysis of transcriptomics and metabolomics provides a robust strategy for deciphering the complex molecular mechanisms underlying drought resistance. In watermelon, integrated time-course omics identified a 6-hour window as a critical response period, revealing adaptation through the co-regulation of starch and sucrose metabolism, hormone signaling, and photosynthetic systems (Chen et al., 2024). Similarly, in *Lilium oriental*, combined omics demonstrated that drought impairs carbohydrate metabolism, and candidate genes identified in these pathways provide targets for genetic engineering and molecular breeding (Cui et al., 2023).

*Pinus tabuliformis* Carrière is widely distributed across the warm-temperate semi-humid to semi-arid zones of Northern, Northwestern, and Northeastern China, serving as a vital species for afforestation. This species is characterized by a well-developed root system and high drought tolerance, performing essential functions in soil and water conservation. However, 1-year-old seedlings are frequently utilized in practical afforestation (Shao et al., 2022). Due to immature root development and physiological regulatory mechanisms, these seedlings exhibit high sensitivity to water deficit. Combined with insufficient post-planting management, drought stress often leads to high mortality rates, significantly limiting the effectiveness of afforestation projects and causing substantial economic and ecological losses.

The broad geographic distribution of *P. tabuliformis* spans significant precipitation and temperature gradients, which has fostered substantial genetic differentiation among populations. Molecular evidence from RAPD markers indicates that significant divergence exists among different seed sources, shaped by both geographic distance and water-temperature conditions (Wang and Gao, 2009). These genetic structures are often reflected in physiological performance, as long-term evolutionary adaptation drives the functional heterogeneity observed across its range (Wenbin. et al., 2018). Previous provenance trials further suggest that populations from arid or marginal habitats frequently evolve specialized adaptive traits, such as enhanced water-use efficiency, to survive local environmental filters (Wang and Hao, 2010). However, the molecular underpinnings, particularly the coordinated transcriptomic and metabolomic shifts that differentiate these provenances during water stress, remain to be fully elucidated. To elucidate drought resistance mechanisms across different geographical environments, two representative provenances were selected: Heilihe (HLH) from Chifeng, Inner Mongolia, and Shangluo (SL) from Shaanxi Province. Current research on drought adaptation in *P. tabuliformis* is largely restricted to aerial tissues, often neglecting the dynamic coordination and strategic differences between the underground root system and needles under prolonged stress. Therefore, an integrated time-course transcriptomic and metabolomic analysis was conducted on both roots and needles across the entire process of drought and rehydration. Functional enrichment analysis of key differentially expressed genes (DEGs), metabolites (DEMs), and trend clusters was performed to decipher organ-and provenance-specific drought resistance mechanisms and to identify key candidate genes and metabolites for drought tolerance in *P. tabuliformis.*

## 2 Materials and methods

### 2.1 Plant materials and drought treatment

*Pinus tabuliformis* seedlings from two provenances, Heilihe (HLH) in Chifeng, Inner Mongolia, and Shangluo (SL) in Shaanxi Province, were selected as the experimental materials. All seedlings met the following criteria: 6–8 months old, 15–20 cm in height, 2–3 mm in ground diameter, free from pests, diseases, and mechanical damage, and exhibiting consistent growth vigor. The experiment consisted of a drought treatment group and a control group. Seedlings in the control group were watered regularly to maintain a soil water content of 80%–85%. In the drought treatment group, irrigation was withheld from the beginning of the experiment. Soil relative water content (SRWC) was monitored throughout the process, reaching approximately 60%, 40%, and 25% at D10, D18, and D26, respectively. After the sampling on Day 26, the remaining seedlings in the drought group were re-watered to field capacity. Leaf and root samples were harvested at five time points for the HLH provenance: Day 10 (D10), Day 18 (D18), Day 26 (D26), Day 27 (R1), and Day 36 (R10). For the SL provenance, sampling was focused on four representative stages (D10, D26, R1, and R10) to optimize the consistency of plant materials and ensure three robust biological replicates for each group. Consequently, the study was structured into four experimental subsets: HLH-Leaf, HLH-Root, SL-Leaf, and SL-Root. At each time point, three biological replicates were collected. All samples were immediately frozen in liquid nitrogen and stored at -80 °C (Thermo Scientific™ Forma 900 Series) for subsequent physiological assays and RNA extraction.

### 2.2 Measurement of physiological indices

The activities of peroxidase (POD) and catalase (CAT), as well as the content of malondialdehyde (MDA), were assayed using commercial kits (Beijing Solarbio Science & Technology Co., Ltd., Beijing, China) following the manufacturer’s protocols. Briefly, 0. 1 g of fresh leaf tissue was homogenized in an ice bath with 1 mL of the corresponding extraction buffer. The homogenate was centrifuged at 8000 × *g* for 10 min at 4°C, and the supernatant was collected for analysis. MDA content was determined using the thiobarbituric acid (TBA) method by mixing the supernatant with TBA working solution. The mixture was boiled at 100°C for 60 min, cooled on ice, and centrifuged. Absorbance was measured at 532 nm and 600 nm to calculate the content. CAT activity was determined by monitoring the decomposition of H_2_O_2_ at 240 nm. The reaction mixture consisted of 0. 1 mL enzyme extract and 2. 9 mL buffer containing H_2_O_2_. One unit (U) of CAT activity was defined as the degradation of 1 μmol H_2_O_2_ per minute per gram of tissue. POD activity was measured using the guaiacol method by recording the absorbance change at 470 nm over 1 min in the presence of H_2_O_2_. One unit (U) of POD activity was defined as an absorbance change of 0. 01 per minute per gram of tissue (Spitz and Oberley, 1989; Minami and Yoshikawa, 1979; Doerge et al., 1997; Johansson and Borg, 1988; Zhou et al., 2024; Pervaiz et al., 2021). The Leaf Relative Water Content (LRWC) was measured using the saturated weighing method. Four to five leaves were randomly selected per replicate and weighed immediately to obtain the fresh weight (FW). The leaves were then immersed in distilled water for 24 h to reach full turgidity. After blotting excess surface water, the turgid weight (TW) was recorded. Finally, the samples were oven-dried at 80 °C for 3 h until a constant weight was achieved to obtain the dry weight (DW). LRWC was calculated using the formula below: RWC(%) = (FW-DW)/(TW-DW)×100%.

### 2.3 Transcriptome library construction and sequencing

Total RNA was treated to remove ribosomal RNA (rRNA) using the rRNA depletion kit, followed by fragmentation in fragmentation buffer. First-strand cDNA was synthesized using random hexamer primers and reverse transcriptase. Second-strand cDNA was subsequently synthesized using dUTP instead of dTTP to ensure strand specificity. The synthesized cDNA fragments underwent end-repair and A-tailing, followed by the ligation of sequencing adapters. The uracil-N-glycosylase (UNG) enzyme was then used to degrade the second-strand cDNA containing dUTP. The remaining first-strand cDNA was enriched by PCR amplification. The PCR products were heat-denatured into single-stranded DNA (ssDNA) and circularized with splint oligos and DNA ligase to form single-strand circular DNA (ssCir DNA). The ssCir DNA was amplified by rolling circle amplification (RCA) to generate DNA Nanoballs (DNBs). Finally, the libraries were sequenced on the DNBSEQ platform (BGI-Shenzhen, China) to generate paired-end reads.

### 2.4 Transcriptomic data processing and differential expression analysis

Raw paired-end reads were quality-controlled using fastp (version 0.23.2) (Chen et al., 2018) with default parameters to remove adapters, poly-N sequences, and low-quality bases. The obtained high-quality clean reads were aligned to the *Pinus tabuliformis* reference genome (version 1. 0) (Niu et al., 2022) using HISAT2 (Kim et al., 2019). Gene expression levels were quantified as Fragments Per Kilobase of exon model per Million mapped fragments (FPKM). Differential expression analysis was performed using the DESeq2 (version 1.48.2) package in R (version 4.5.1) (Love et al., 2014). Genes with an adjusted *P*-value (*Padj*) < 0. 05 and an absolute log2 fold change (*|*log_2_FC|) > 1 were identified as differentially expressed genes (DEGs). To elucidate the potential functions of key hub genes that were unannotated in the reference genome, their coding sequences (CDS) were extracted and aligned against the NCBI non-redundant (nr) protein database using BLASTX (E-value ≤ 1.0 × 10^–5^) based on sequence homology.

### 2.5 Metabolite extraction and LC-MS/MS analysis

Metabolites were extracted using a mixture of methanol, acetonitrile, and water (2:2:1, v/v/v) containing internal standards. After homogenization and sonication, samples were incubated at -20 °C for 1 h to precipitate proteins and centrifuged to collect the supernatant. Analysis was performed on a Vanquish ultra-high-performance liquid chromatography (UHPLC) system coupled with an Orbitrap Exploris 120 mass spectrometer (Thermo Fisher Scientific, San Jose, CA, USA) operated under the control of Xcalibur software (version 4.4, Thermo Fisher Scientific). Chromatographic separation was achieved on an ACQUITY UPLC BEH Amide column (2.1 mm × 50 mm, 1.7 μm). Mobile phase A was an aqueous solution of 25 mmol/L ammonium acetate and 25 mmol/L ammonia water, and mobile phase B was acetonitrile. The separation was performed via a gradient elution at a flow rate of 400 μL/min. Mass spectra were acquired in both positive and negative modes (scan range 70–1050 m/z) (Sumner et al., 2007; Wang et al., 2016; Zhou et al., 2022).

### 2.6 Metabolomic data processing and differential accumulation analysis

The raw mass spectrometry data (.raw) were converted to the mzXML format using ProteoWizard (version 3.0.24054) (Adusumilli and Mallick, 2017). Metabolite identification and data preprocessing were performed using a specialized R-based pipeline integrated with an in-house standard database and the BT-Plant database (version 1.1). A total of 32,900 metabolic features were extracted. Multivariate statistical analyses, including Orthogonal Partial Least Squares Discriminant Analysis (OPLS-DA), were conducted using R (version 4.5.1). Differentially accumulated metabolites (DAMs) were screened based on a Variable Importance in Projection (VIP) score > 1, a P-value < 0. 05 (Student’s t-test), and a fold change (FC) > 1. 5.

### 2.7 Time-series clustering and functional enrichment analysis

To decipher the temporal dynamics of drought responses, the expression profiles of DEGs and DAMs were first standardized via Z-score transformation. Soft clustering was performed using the Fuzzy C-Means algorithm implemented in the Mfuzz package (version 2.68.0) within the R environment (version 4.5.1), where genes and metabolites exhibiting synchronized temporal profiles were partitioned into discrete clusters according to their membership scores (Futschik and Carlisle, 2005). For functional interpretation, distinct strategies were applied to the transcriptomic and metabolomic datasets. For DEGs, the whole-genome protein sequences of *P. tabuliformis* were first submitted to the eggNOG-mapper online server (http://eggnog-mapper.embl.de/) to generate comprehensive functional annotations (Cantalapiedra et al., 2021). Based on this background, Gene Ontology (GO) and KEGG pathway enrichment analyses were performed using the clusterProfiler package (version 4.16.0) in R (version 4.5.1) (Wu et al., 2021). For DAMs, KEGG pathway enrichment analysis was conducted using the MetaboAnalyst 5.0 (Pang et al., 2021)web platform (https://www.metaboanalyst.ca/). The resulting enriched pathways were filtered based on statistical significance (*P* < 0. 05), and the visualization of enrichment results for both datasets was performed using custom R scripts.

### 2.8 Construction of gene-metabolite co-expression networks

To integrate transcriptomic and metabolomic data, Pearson correlation coefficients (PCC) were calculated between DEGs and DAMs using R (version 4.5.1). To ensure high confidence, a strict threshold was applied: |r| > 0. 8 and P < 0. 05. Furthermore, a Top-k filtering strategy (k=20) was adopted to retain the strongest regulatory connections for each hub metabolite, thereby reducing visual redundancy. The resulting networks were visualized using Cytoscape 3.10.4 software (Shannon et al., 2003).

### 2.9 Weighted gene co-expression network analysis

Weighted gene co-expression networks were constructed using the WGCNA package (version 1.73) in R (version 4.5.1). Transcriptome data were pre-processed by retaining the top 18,000 genes with the highest variance (Log2-transformed). A signed network was constructed using the blockwiseModules function with a soft-thresholding power of β=10 to satisfy the scale-free topology criterion. Co-expression modules were identified using the Dynamic Tree Cut algorithm (deepSplit=3, minModuleSize=20). Modules with high similarity (dissimilarity < 0. 20, correlation > 0. 80) were merged. Module-trait associations were assessed by calculating Pearson correlations between module eigengenes (MEs) and binary traits or metabolite abundances. Significant modules were identified based on |r| > 0. 5 and P < 0. 05 (Langfelder and Horvath, 2008; Zhang and Horvath, 2005).

## 3 Results

### 3.1 Impact of drought on physiological indicators of *Pinus tabuliformis*

**Figure 1:**
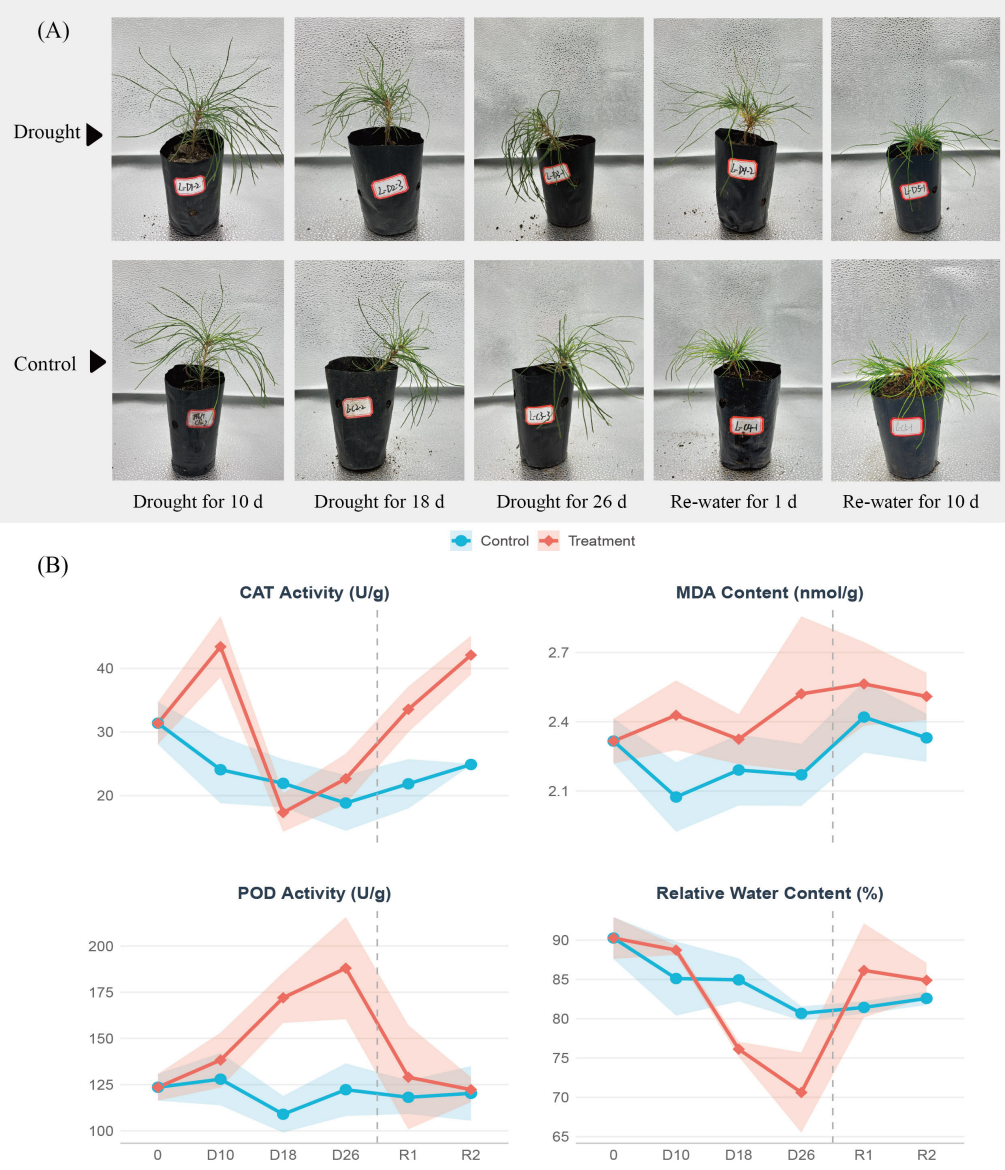
Phenotypic and physiological indices measurements of drought in *Pinus tabuliformis.***(A)** Phenotypes in mild drought (10d), severe drought (26 d), and after rewater(1d,10d). **(B)** Leaf relative water content and enzymatic activity indicators.

Under drought treatment, *Pinus tabuliformis* exhibited mild, moderate, and severe drought stress on days 10, 18, and 26, respectively (**Figure 1A**). Physiological indicators, including peroxidase (POD), catalase (CAT), and the lipid peroxidation product malondialdehyde (MDA), showed distinct responses to water deficit. In this study, antioxidant enzyme activities and leaf relative water content (RWC) were quantified. It was observed that POD activity remained significantly higher than the control throughout the entire duration of drought stress (**Figure 1B**). CAT activity was significantly elevated during mild drought but became inhibited under severe drought conditions; however, it exhibited a rapid rebound post-rehydration, eventually exceeding control levels. Following rehydration, RWC quickly returned to levels comparable with the control group. In contrast, MDA levels exhibited no significant variation during mild drought but increased under severe drought, indicating oxidative damage to cellular membranes. Overall, antioxidant enzyme activities remained generally higher than the control or demonstrated rapid recovery during both the drought and rehydration phases. These results suggest that these enzymes play a critical role in the drought resistance and post-stress recovery of the HLH provenance.

### 3.2 Global transcriptomic response of *Pinus tabuliformis* to drought

**Figure 2:**
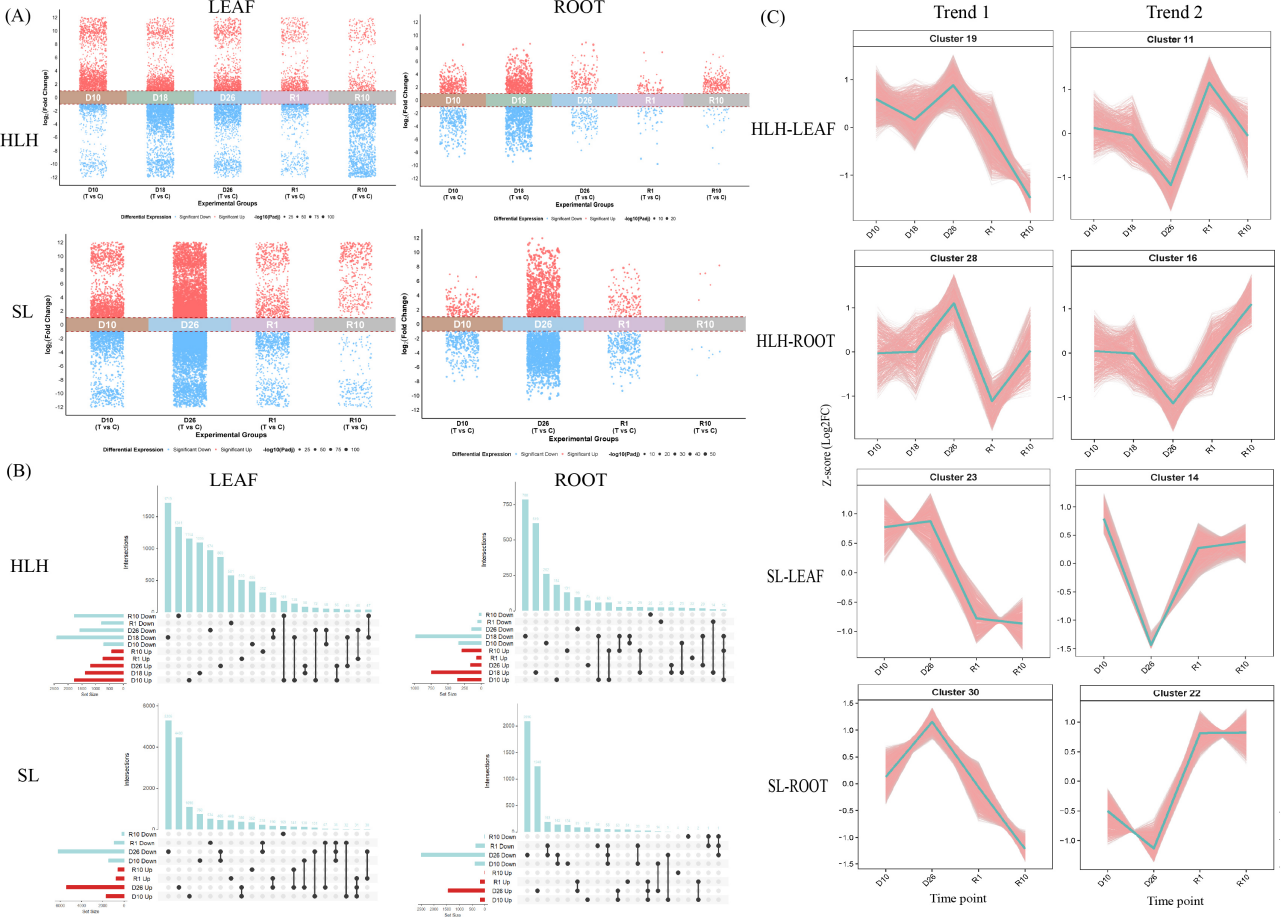
Global transcriptomic response to drought in *Pinus tabuliformis*. **(A)** Volcano plots of differentially expressed genes (DEGs) in leaves and roots of HLH and SL provenances. **(B)** UpSet plots showing the intersections of DEGs across different drought and rewatering stages. **(C)** Time-series clustering analysis of gene expression profiles. Two representative patterns are shown: Trend1(up-regulated during drought, down-regulated upon rewatering) and Trend2 (down-regulated during drought, up-regulated upon rewatering).

To investigate the gene expression profiles of *Pinus tabuliformis* HLH and SL provenances under drought stress, transcriptomic sequencing was performed on both drought-treated and control (normally watered) samples. Genes were analyzed across four groups: HLH-Root, HLH-Leaf, SL-Root, and SL-Leaf. Based on the filtering criteria of Padj < 0. 05 and |log2 (fold change)| > 1, the distribution of differentially expressed genes (DEGs) across different drought stages was determined (**Figure 2A**; **Supplementary Table 1**), and comparisons were made between the treatment and control groups at each time point (**Figure 2B**). In HLH-Leaf, the number of DEGs for D10, D18, D26, R1, and R10 relative to their respective controls was 2,493 (1,782 upregulated and 711 downregulated), 3,791 (1,374 upregulated and 2,417 downregulated), 2,773 (1,197 upregulated and 1,576 downregulated), 1,534 (743 upregulated and 791 downregulated), and 2,202 (427 upregulated and 1,775 downregulated), respectively. In HLH-Root, the identified DEGs were 695 (355 upregulated and 340 downregulated), 1,734 (746 upregulated and 988 downregulated), 308 (163 upregulated and 145 downregulated), 129 (73 upregulated and 56 downregulated), and 324 (289 upregulated and 35 downregulated). For the SL provenance, SL-Leaf exhibited 3,180 DEGs (1,709 upregulated and 1,471 downregulated) at D10, 11,672 (5,441 upregulated and 6,231 downregulated) at D26, 1,723 (769 upregulated and 954 downregulated) at R1, and 805 (574 upregulated and 231 downregulated) at R10. In SL-Root, the DEGs were 572 (186 upregulated and 386 downregulated), 3,944 (1,441 upregulated and 2,503 downregulated), 535 (179 upregulated and 356 downregulated), and 12 (5 upregulated and 7 downregulated) at the corresponding time points.

The number of DEGs identified in leaves across all time points was significantly higher than that in roots, indicating a more pronounced transcriptomic response in the aerial tissues. Distinct temporal characteristics in gene expression were observed between the two provenances. The HLH provenance showed fewer shared DEGs between different stress stages; specifically, only 320 DEGs were shared between D18 and D26 in leaves, and 114 were shared between D10 and D26. In HLH roots, only 29 DEGs were shared between D10 and D18, and 25 between D18 and D26. In contrast, the SL provenance exhibited a higher degree of continuous differential expression across stages. In SL leaves, 845 genes were differentially expressed across both drought periods, with 69 genes maintaining differential expression through D10, D26, and the first day of rehydration (R1). In SL roots, 198 genes were shared during the drought period, while 91 genes maintained their status from drought through the rehydration stage (Figure. 2B). These data suggest that compared to the HLH provenance, the SL provenance maintains more persistent transcriptomic alterations during drought and rehydration.

Time-course clustering analysis was performed for the four groups (HLH-Root, HLH-Leaf, SL-Root, and SL-Leaf), resulting in 30 clusters per group. Two representative trends were identified (**Figure 2C**). Trend 1, characterized by an increase in expression during drought followed by an immediate decrease upon rehydration, was observed in Cluster 28 (HLH-Root), Cluster 19 (HLH-Leaf), Cluster 30 (SL-Root), and Cluster 23 (SL-Leaf). These genes indicate a continuous response during the drought stages. Trend 2, showing a decrease during drought followed by recovery after rehydration, was identified in Cluster 16 (HLH-Root), Cluster 11 (HLH-Leaf), Cluster 14 (SL-Leaf), and Cluster 22 (SL-Root).

### 3.3 Global metabolomic response of *Pinus tabuliformis* to drought

**Figure 3:**
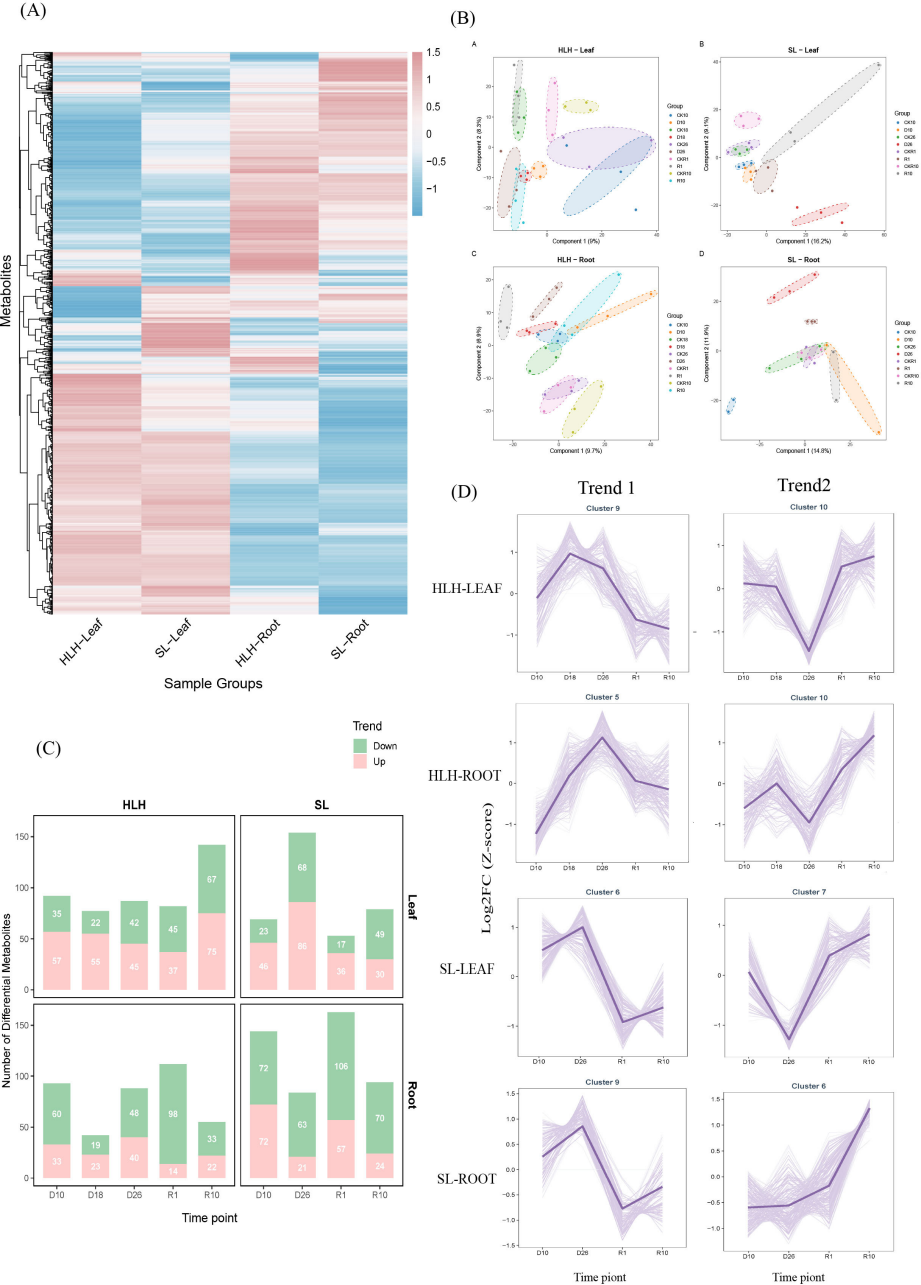
Global metabolic response to drought in *Pinus tabuliformis*. **(A)** Heatmap clustering analysis of metabolite expression patterns across different tissues and provenances. **(B)** Partial least squares-discriminant analysis (PLS-DA) score plots showing the separation between control and drought-treated samples. **(C)** Statistical summary of differentially accumulated metabolites (DAMs) at each treatment stage. **(D)** Time-series clustering analysis of metabolite profiles. Two representative patterns consistent with the transcriptomic trends are shown: Trend 1 (up-regulated during drought, down-regulated upon rewatering) and Trend 2 (down-regulated during drought, up-regulated upon rewatering).

To gain a comprehensive understanding of the metabolic dynamics in *Pinus tabuliformis* under drought stress, untargeted metabolomics was employed to analyze both root and needle tissues. Hierarchical clustering analysis, visualized via a heatmap (**Figure 3A**), revealed distinct metabolic expression patterns between different tissues and provenances. Partial least squares-discriminant analysis (PLS-DA) demonstrated a distinct separation between the control (CK) and drought-treated groups (**Figure 3B**), indicating that significant metabolic alterations occurred across varying drought intensities. Differentially accumulated metabolites (DAMs) were screened based on the criteria of variable importance in projection (VIP) > 1, P < 0. 05, and a fold change threshold > 1. 5. The distribution of DAMs at different stages is presented in **Figure 3C**. A total of 1,160 DAMs were identified across the study (**Supplementary Table 2**). The number of DAMs per treatment group exhibited considerable variation, ranging from 14 to 106. During the severe drought stage (D26), 87 significant DAMs (45 upregulated and 42 downregulated) were identified in HLH leaves, whereas 154 significant DAMs (86 upregulated and 68 downregulated) were found in SL leaves (**Figure 3C**). These results indicate a significant difference in the metabolic response intensity to water stress between the two provenances.

Time-course clustering analysis was performed for all metabolites, resulting in 12 distinct accumulation patterns for each group, which highlighted two characteristic trends (**Figure 3D**). Trend 1, characterized by elevated metabolite accumulation during the drought stages followed by a decline upon rehydration, was observed in Cluster 9 (HLH-Leaf), Cluster 5 (HLH-Root), Cluster 6 (SL-Leaf), and Cluster 9 (SL-Root). Conversely, Trend 2, showing a decrease in abundance during drought and an increase following rehydration, was identified in Cluster 10 (HLH-Leaf), Cluster 10 (HLH-Root), Cluster 7 (SL-Leaf), and Cluster 6 (SL-Root).

### 3.4 GO enrichment analysis of differentially expressed genes

**Figure 4:**
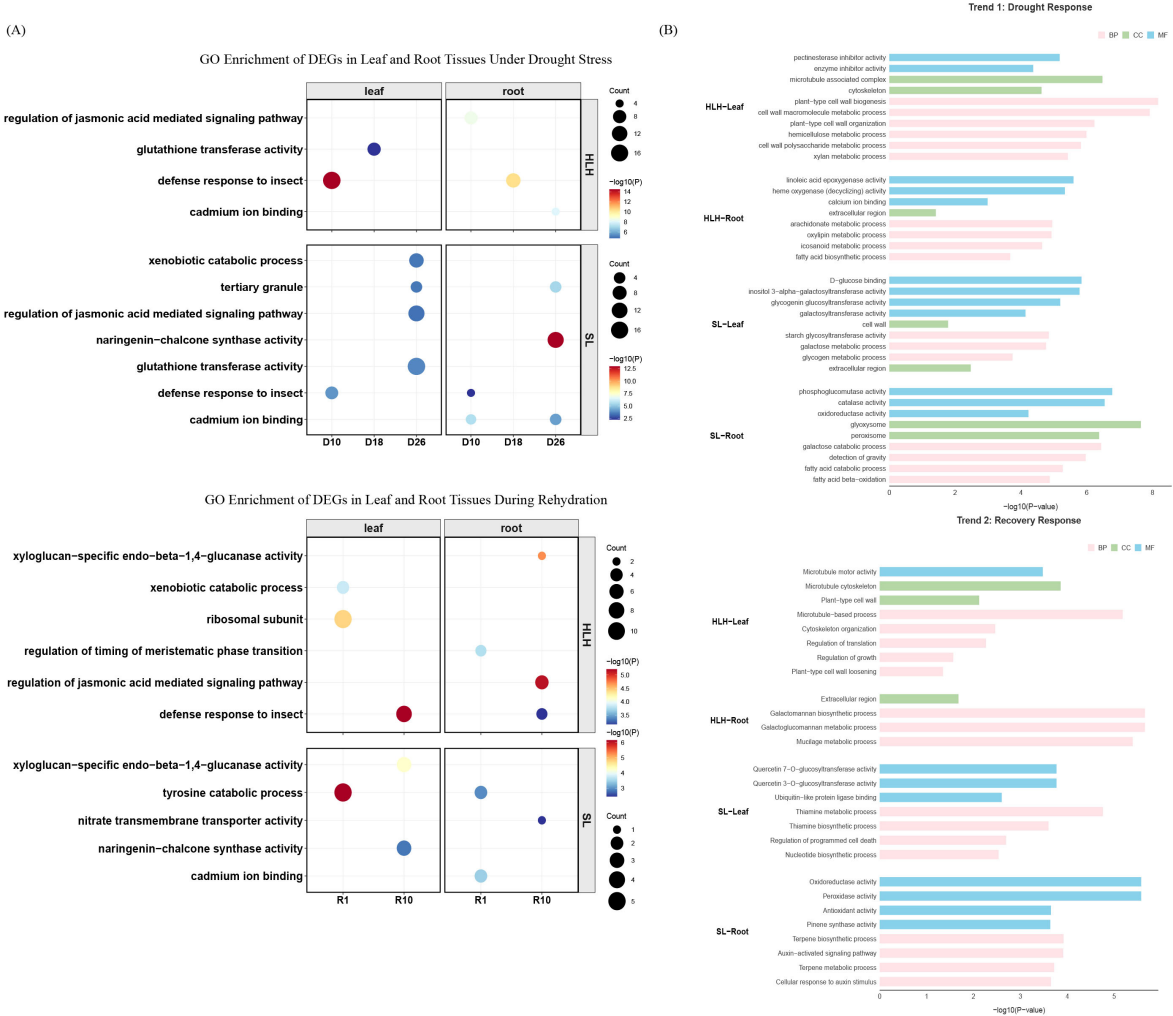
Gene Ontology (GO) enrichment analysis of gene expression in *Pinus tabuliformis* under drought and rewatering. **(A)** GO enrichment analysis of differentially expressed genes (DEGs) across different drought and rewatering stages in leaves and roots of HLH and SL provenances. **(B)** GO enrichment analysis of genes belonging to the representative temporal patterns identified in **Figure 2C**: GO enrichment analysis of Trend 1 (up-regulated during drought, down-regulated upon rewatering) and Trend 2 (down-regulated during drought, up-regulated upon rewatering).

To elucidate the post-transcriptional regulatory divergence between the HLH and SL provenances, Gene Ontology (GO) functional enrichment analysis was performed on identified DEGs (**Supplementary Tables 4**, **5**). While shared core stress pathways were observed, the two provenances exhibited distinct adaptive strategies regarding resource allocation and temporal response. The HLH provenance employed a signal-driven plasticity strategy for rapid recovery, whereas the SL provenance exhibited a highly dynamic response focused on structural defense and homeostasis maintenance (**Figure 4A**).

During the water deficit phase, the primary defense mechanisms differed fundamentally between the provenances. The HLH response was characterized by a systemic signal-driven mechanism that persisted throughout the early and middle stages of drought (**Supplementary Table 4**). On days 10 and 18 (D10 and D18), the root tissues of HLH were significantly enriched in the regulation of jasmonic acid mediated signaling pathway and defense response to insect. This indicates that the underground tissues prioritized the activation of Jasmonic Acid-centered systemic defense signals. Concurrently, the upregulation of glutathione transferase activity in HLH needles suggested that aerial tissues were primarily dedicated to scavenging drought-induced reactive oxygen species (ROS) to maintain cellular redox balance (**Figure 4A**).

Conversely, the SL provenance relied more heavily on secondary metabolites and physical barriers. At D26, SL roots exhibited highly significant enrichment in naringenin-chalcone synthase activity, a key precursor for flavonoid biosynthesis, suggesting the construction of a chemical defense system. Simultaneously, SL needles were enriched in genes related to tertiary granule (**Supplementary Table 5**), which are involved in the secretion of cell wall remodeling enzymes, indicating active physical barrier reconstruction to minimize water loss. Additionally, the enrichment of cadmium ion binding in SL roots at D10 may reflect a specialized regulatory mechanism for metal ion homeostasis, potentially playing a unique role in maintaining osmotic potential.

The rehydration phase further highlighted the divergent recovery mechanisms of the two provenances. The HLH provenance demonstrated an vigorous growth-restart capability. At R1, HLH needles showed highly significant induction of pathways related to xenobiotic catabolic process and ribosomal subunit. The large-scale upregulation of ribosomal components provides molecular evidence for the release of metabolic inhibition and the initiation of growth programs. Correspondingly, HLH roots activated the regulation of timing of meristematic phase transition, confirming the restoration of meristematic activity to compensate for biomass loss. In contrast, the recovery of the SL provenance was more steady but lagged. At R1, SL needles maintained high tyrosine catabolic process activity rather than initiating protein synthesis. By R10, nitrate transmembrane transporter activity in roots and xyloglucan-specific endo-beta-1,4-glucanase activity in needles became active. This suggests metabolic inertia in the SL provenance, where structural repair and nutrient uptake were prioritized over rapid growth post-rehydration. In summary, the HLH provenance adopted a high-input, high-plasticity strategy, achieving rapid recovery through intensive hormone signaling and efficient protein synthesis. Conversely, the SL provenance prioritized a highly dynamic defense, utilizing secondary metabolic defenses and structural reinforcement, resulting in a more robust but gradual recovery process.

### 3.5 GO functional enrichment analysis based on temporal clustering

To further characterize the biological processes underlying distinct expression profiles, GO enrichment analysis was performed on specific gene clusters exhibiting representative temporal trends (**Figure 4B**). First, genes including Trend1 (upregulated during drought and immediately downregulated upon rehydration) were analyzed. In HLH-Leaf (Cluster 19), DEGs were predominantly enriched in pathways related to cell wall biosynthesis and structural reinforcement, including plant-type secondary cell wall biogenesis, hemicellulose metabolic process, and xylan metabolic process. In HLH-Root (Cluster 28), enrichment was observed in oxylipin metabolism and fatty acid biosynthesis, specifically linoleic acid epoxygenase activity, oxylipin metabolic process, and long-chain fatty acid biosynthetic process. Conversely, SL-Root (Cluster 30) showed significant enrichment in lipid catabolic process and energy metabolism, including glyoxysome, fatty acid beta-oxidation, and phosphoglucomutase activity. In SL-Leaf (Cluster 23), genes were primarily involved in the biosynthesis of osmoprotectants and stress-responsive oligosaccharides, such as inositol 3-alpha-galactosyltransferase activity (involved in raffinose family oligosaccharide synthesis), D-glucose binding, and glycogen biosynthetic process. These results indicate that the two provenances employ divergent physiological response mechanisms under water deficit. The HLH provenance primarily enhances physical structural barriers, as evidenced by the upregulation of secondary cell wall biogenesis in leaves to maintain mechanical strength and the promotion of long-chain fatty acid synthesis in roots to establish suberized barriers. In contrast, the SL provenance relies more on biochemical metabolic adjustments to maintain cellular homeostasis; leaves synthesize osmoprotectants through carbohydrate metabolic pathways, while roots activate fatty acid β-oxidation to supplement energy supply through lipid degradation, suggesting a state of energy metabolic deficit under stress.

Subsequently, GO enrichment was performed on genes including Trend 2 (downregulated during drought and rapidly upregulated upon rehydration). In HLH-Root (Cluster 16), pathways related to cell wall polysaccharide biosynthesis were enriched, including galactoglucomannan metabolic process, galactomannan biosynthetic process, and mucilage metabolic process. Similarly, HLH-Leaf (Cluster 11) was enriched in cell wall biogenesis and metabolism and cytoskeleton organization, specifically plant-type secondary cell wall biogenesis, hemicellulose metabolic process, and microtubule-based movement. For the SL provenance, SL-Root (Cluster 22) was primarily enriched in secondary metabolite biosynthesis and antioxidant enzyme activity, including peroxidase activity, monoterpenoid metabolic process, and pinene synthase activity. Finally, SL-Leaf (Cluster 14) showed enrichment in vitamin metabolism and secondary metabolite modification, such as thiamine metabolic process, thiamine biosynthetic process, and quercetin O-glucosyltransferase activity. Following rehydration, the HLH provenance demonstrated a rapid initiation of cell wall polysaccharide synthesis and mucilage secretion, indicative of robust growth rebound and tissue repair capabilities. Conversely, the SL provenance prioritized the upregulation of secondary metabolite synthesis, antioxidant enzymes, and coenzyme metabolism, suggesting a phase of defense system repair and metabolic network restoration. This divergence suggests that the HLH provenance possesses superior resilience, whereas the SL provenance adopts a recovery strategy focused on the re-establishment of homeostasis.

### 3.6 KEGG pathway enrichment analysis based on differentially expressed genes

**Figure 5:**
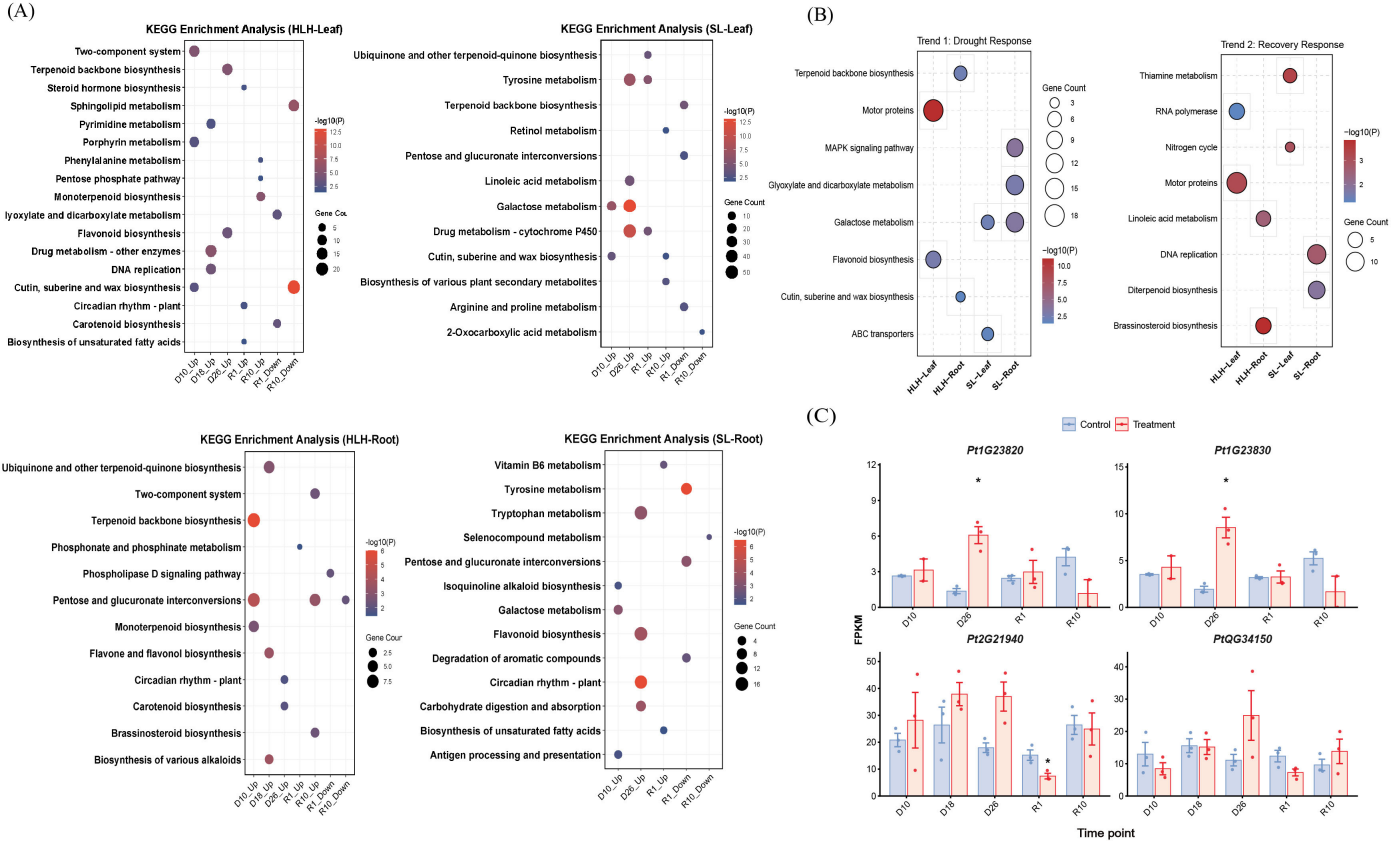
Kyoto Encyclopedia of Genes and Genomes (KEGG) pathway enrichment analysis of gene expression in *Pinus tabuliformis* under drought and rewatering. **(A)** KEGG pathway enrichment analysis of differentially expressed genes (DEGs) across different drought and rewatering stages in leaves and roots of HLH and SL provenances. **(B)** KEGG pathway enrichment analysis of genes belonging to the representative temporal patterns identified in **Figure 2C**: Trend 1 (up-regulated during drought, down-regulated upon rewatering) and Trend 2 (down-regulated during drought, up-regulated upon rewatering). **(C)** FPKM of Key gene.

To elucidate the metabolic regulatory mechanisms at the transcriptional level during drought stress and rewatering recovery in HLH and SL provenances, KEGG pathway enrichment analysis was performed on differentially expressed genes (DEGs) from each comparison group (**Figure 5A**).

In leaves of the HLH provenance, DEGs were predominantly enriched in pathways related to environmental signal perception and structural barrier formation. At the initial drought stage (10 days of drought), the two-component system and cutin, suberine and wax biosynthesis pathways were significantly upregulated, indicating rapid activation of signal transduction and reinforcement of the epidermal barrier to reduce water loss early in the stress response. As drought intensity increased (26 days of drought), the flavonoid biosynthesis and terpenoid backbone biosynthesis pathways were significantly enriched, suggesting that secondary metabolite accumulation is a key strategy for coping with prolonged oxidative stress. During rewatering (1 day of rewatering), the circadian rhythm - plant pathway was activated, reflecting the re-establishment of photoperiod-related growth rhythms. In contrast, the response in roots of the HLH provenance focused more on secondary metabolic defense. At the early drought stage, terpenoid backbone biosynthesis and pentose and glucuronate interconversions were significantly active. By 18 days of drought, flavone and flavonol biosynthesis and the biosynthesis of various alkaloids were significantly upregulated, indicating that roots adapt to soil water deficit by modulating their secondary metabolic profile.

The SL provenance exhibited response characteristics primarily centered on the regulation of carbon and nitrogen metabolism. In leaves of the SL provenance, galactose metabolism was significantly enriched at both early (10 days) and late (26 days) drought stages, with a high number of enriched genes, indicating that regulating soluble sugar metabolism to maintain cellular osmotic potential is a key mechanism for SL in response to drought. Furthermore, prolonged drought (26 days) induced the expression of pathways for drug metabolism - cytochrome P450, tyrosine metabolism, and linoleic acid metabolism, implying the activation of detoxification enzymes and remodeling of lipid and amino acid metabolism play important roles in stress resistance. At the initial rewatering stage (1 day of rewatering), ubiquinone and other terpenoid-quinone biosynthesis was significantly upregulated, potentially involved in restoring electron transport chain function. Roots of the SL provenance also showed an early response in galactose metabolism. However, under prolonged drought stress (26 days), flavonoid biosynthesis, circadian rhythm - plant, and tryptophan metabolism emerged as core response pathways. This aligns with the upregulation of naringenin chalcone synthase activity found in the GO analysis, confirming that roots build a chemical defense system by synthesizing flavonoids under long-term stress. After rewatering (1 day of rewatering), vitamin B6 metabolism and the biosynthesis of unsaturated fatty acids were significantly enriched, suggesting that root cells prioritize coenzyme synthesis and membrane lipid system repair during the recovery phase.

### 3.7 KEGG pathway enrichment analysis based on temporal gene expression profiles

To further elucidate the dynamic regulatory mechanisms during drought and rehydration, KEGG enrichment was performed on representative gene trends (**Figure 5B**). For Trend 1 (upregulated during drought), HLH and SL exhibited divergent adaptive strategies. HLH focused on structural and chemical defenses; needles were enriched in Flavonoid biosynthesis and Motor proteins, while root tissues upregulated Cutin, suberine and wax biosynthesis and Terpenoid backbone biosynthesis, signaling the formation of hydrophobic barriers and defensive compounds. Conversely, SL prioritized signaling and osmotic regulation. SL roots exhibited enriched MAPK signaling pathway and Glyoxylate and dicarboxylate metabolism, suggesting lipid-derived energy mobilization. The concurrent upregulation of Galactose metabolism and ABC transporters in both tissues underscores the role of osmoprotectant transport in SL homeostasis.

Regarding Trend 2 (downregulated during drought), rehydration triggered distinct growth restoration pathways. HLH showed prominent growth re-initiation and membrane repair, evidenced by enriched RNA polymerase and Motor proteins in needles, and Brassinosteroid biosynthesis and Linoleic acid metabolism in roots. In contrast, SL prioritized genetic replication and basal metabolism, characterized by enriched DNA replication and Diterpenoid biosynthesis in roots, alongside active Nitrogen cycle and Thiamine metabolism in needles to restore photosynthetic efficiency. In summary, HLH adopts an active plasticity strategy utilizing hormone-driven compensatory growth, whereas SL employs a highly dynamic defense-repair mechanism centered on signaling, osmotic networks, and the restoration of basal metabolic potential.

To further refine the key genes identified from enriched pathways, we examined their expression dynamics (**Figure 5C**). In the roots of the HLH provenance, the expression levels of *PtDXS1* (Pt2G21940), involved in terpenoid backbone biosynthesis, and *PtCLO* (PtQG34150), associated with cutin synthesis, were significantly higher under drought stress and early rehydration (D10–R1) compared to the control (*P* < 0. 05), indicating that the corresponding biosynthetic pathways were notably activated by stress. Meanwhile, in the roots of the SL provenance, the expression of catalase genes *PtCAT1*(Pt1G23820) and *PtCAT2*(Pt1G23830) was significantly up-regulated under drought stress, with particularly higher levels during severe drought or rehydration phases compared to the control.

### 3.8 KEGG pathway enrichment analysis of differentially accumulated metabolites

**Figure 6:**
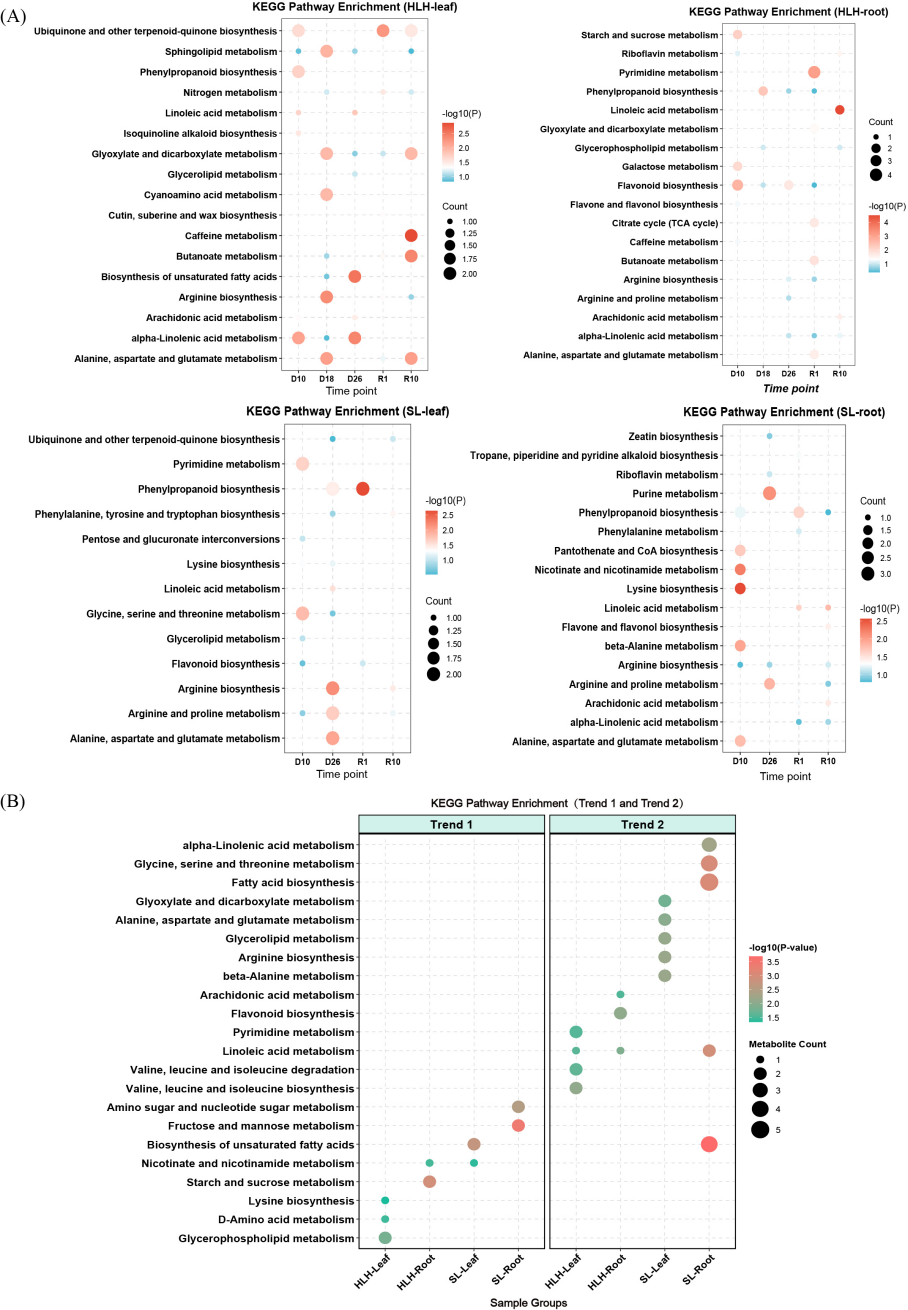
Kyoto Encyclopedia of Genes and Genomes (KEGG) pathway enrichment analysis of metabolic changes in *Pinus tabuliformis* under drought and rewatering. **(A)** KEGG pathway enrichment analysis of differentially accumulated metabolites (DAMs) across different drought and rewatering stages in leaves and roots of HLH and SL provenances. **(B)** KEGG pathway enrichment analysis of metabolites belonging to the representative temporal patterns identified in **Figure 3D**: Trend 1 (up-regulated during drought, down-regulated upon rewatering) and Trend 2 (down-regulated during drought, up-regulated upon rewatering).

KEGG enrichment analysis characterized the dynamic reconfiguration of metabolic networks across temporal, tissue, and provenance dimensions (**Figure 6A**). Drought stages primarily induced Arginine and proline metabolism and Biosynthesis of unsaturated fatty acids for turgor maintenance and membrane stability, whereas rehydration shifted toward the Citrate cycle (TCA cycle), Pyrimidine metabolism, and secondary metabolic pathways, signaling a transition from defense to growth restoration. Tissue-specific profiles showed that needles prioritized signaling and membrane protection via alpha-Linolenic acid metabolism, while roots emphasized structural defense through Phenylpropanoid biosynthesis and Starch and sucrose metabolism to reinforce cell walls and regulate osmotic potential. Provenance comparisons revealed a more resilient mechanism in HLH, evidenced by the rapid activation of the TCA cycle and pyrimidine metabolism in roots during early rehydration, whereas SL maintained defensive pathways like Flavonoid biosynthesis, indicating persistent oxidative stress and a lack of rapid growth recovery.

Integrated analysis identified Arginine and proline metabolism, alpha-Linolenic acid metabolism, and Phenylpropanoid biosynthesis as the core drought-responsive pathways in *P. tabuliformis*. Proline synthesis within Arginine and proline metabolism facilitated critical osmotic adjustment and ROS scavenging during severe drought, while alpha-Linolenic acid metabolism and Linoleic acid metabolism mediated membrane stability and Jasmonic acid signaling. Phenylpropanoid biosynthesis and Flavonoid biosynthesis established essential chemical barriers in roots by promoting lignin and antioxidant accumulation to enhance mechanical strength and dehydration tolerance. Furthermore, the root-specific activation of the Citrate cycle in HLH was identified as a key energy metabolic hub determining provenance recovery, serving as a vital metabolic indicator for assessing the drought resilience potential of *P. tabuliformis* provenances.

### 3.9 KEGG pathway enrichment analysis of metabolite clusters based on temporal profiles

The Mfuzz soft clustering algorithm was utilized to categorize metabolites into 12 clusters, focusing on two biologically significant trends (**Figure 6B**): drought-induced accumulation (Trend 1) and rehydration-recovery (Trend 2). During the drought stress stage (Trend 1), divergent metabolic strategies were observed in the roots. HLH roots (Cluster 5) showed significant enrichment in Starch and sucrose metabolism (P < 0. 01), indicating the accumulation of soluble sugars like sucrose for osmotic adjustment to maintain cellular turgor. In contrast, SL roots (Cluster 9) primarily enriched Fructose and mannose metabolism. In needles, Glycerophospholipid metabolism was active in HLH (Cluster 9), while SL (Cluster 6) prioritized the Biosynthesis of unsaturated fatty acids.

During the rehydration-recovery phase (Trend 2), metabolic reprogramming directions diverged. SL roots (Cluster 6) exhibited highly significant enrichment in Fatty acid biosynthesis and Biosynthesis of unsaturated fatty acids (P < 0. 001), suggesting prioritized lipid synthesis for repairing damaged membrane systems. Conversely, HLH roots (Cluster 10) significantly enriched the Flavonoid biosynthesis pathway, reflecting the rapid activation of secondary metabolism for antioxidant defense and growth regulation post-stress. In summary, HLH maintained osmotic balance via sucrose metabolism during drought and activated secondary metabolism upon rehydration, whereas SL demonstrated a pronounced demand for lipid synthesis during recovery, suggesting more extensive membrane repair requirements.

### 3.10 Construction of transcriptome-metabolome correlation networks and identification of drought-responsive hubs

**Figure 7:**
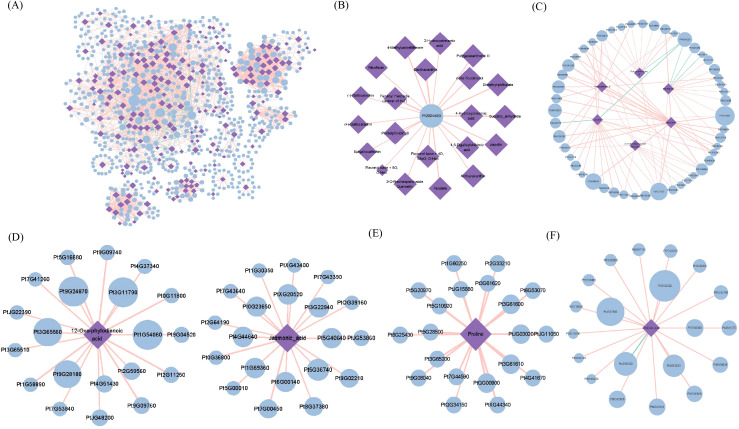
Transcriptome-metabolome co-expression network analysis. **(A)** Global gene-metabolite co-expression network. **(B)** Specific sub-network of the hub gene Pt2G34430. **(C)** Co-expression module of Flavonoids. **(D)** Co-expression module of Jasmonic acid (JA) and OPDA. **(E)** Co-expression module of Shikimic acid. **(F)** Co-expression module of Proline. *Note*: Edges indicate significant Pearson correlations (|*r*| > 0. 8, *P* < 0. 05). Red solid lines represent positive correlations, and green dashed lines represent negative correlations. For clarity, only the top 20 genes with the strongest correlation to each hub metabolite are retained in the sub-networks.

To systematically characterize the transcriptomic-metabolic landscape of *Pinus tabuliformis* under drought, a weighted co-expression network was constructed based on DEGs and DAMs. To ensure high confidence and minimize redundancy, a dual-filtering criterion (Pearson |r| > 0. 8, P < 0. 05) and a Top-20 strategy per metabolite node were applied. The final network comprised 897 nodes and 2,704 edges (**Figure 7A**). Among these, 19 hub genes and 103 hub metabolites (Degree ≥20) were identified. Functional screening based on GO annotations identified Pt2G34430 (Degree = 21) as a central regulator associated with response to water deprivation (GO:0009414). The sub-network centered on Pt2G34430 exhibited broad-spectrum metabolic regulation, showing strong positive correlations with 21 metabolites, including photoprotective carotenoids (antheraxanthin, canthaxanthin), ROS-scavenging flavonoids (gallocatechin, quercetin derivatives), and cell wall-fortifying phenolic acids (ferulate) (**Figure 7B**).

The regulatory architecture of secondary metabolism was characterized by a synergistic activation-repression mechanism. Hub metabolites including flavonoids (Zapotin, Prodelphinidin B), shikimic acid, and jasmonic acid (JA) occupied central topological positions. Pt7G32520 and PtJG27630 were identified as primary transcriptional activators, with PtJG27630 showing an extremely high correlation with Bavachin (PCC = 0. 918). Notably, Pt7G32520 appears to function as a top-level regulator, simultaneously coordinating shikimic acid supply and downstream flavonoid biosynthesis (**Figures 7C, E**). Conversely, Pt4G55220 (Degree = 33) exhibited a broad-spectrum negative correlation with both shikimic acid and various flavonoids, suggesting a feedback inhibition mechanism required to maintain metabolic homeostasis under prolonged stress.

Analysis of the hormone-related sub-network revealed a modular separation between 12-Oxo-phytodienoic acid (OPDA) and Jasmonic acid (JA) regulation (**Figure 7D**). In the OPDA module, Pt9G09760 exhibited high betweenness centrality and a strong correlation with OPDA (PCC = 0. 878), suggesting its role in maintaining the upstream precursor pool via the AOS pathway. In the downstream JA module, Pt0G36800, encoding a vacuolar malate transmembrane transporter, showed the strongest co-expression with JA (PCC = 0. 883). Since malate transport is critical for guard cell turgor regulation, this suggests Pt0G36800 acts as a key effector for JA-induced stomatal closure. Furthermore, the hub gene Pt5G36740, homologous to LURP-one-related proteins, was identified as a central signal integrator. Together, Pt5G36740 and Pt0G36800 constitute a signaling-execution module that translates chemical stress signals into physiological water-saving actions.

Finally, the regulation of Proline accumulation revealed a highly coordinated metabolic-structural defense module (**Figure 7F**). Pt5G10920, showing the highest correlation with proline (PCC = 0. 919), was identified as a homolog of Laccase-12 through BLASTX analysis and GO enrichment (copper ion binding, apoplast). This tight co-regulation reveals a comprehensive adaptation strategy in *P. tabuliformis*: intracellularly, proline accumulation lowers osmotic potential to maintain turgor; extracellularly, laccase-mediated lignification reinforces the cell wall within the apoplast. This synchronization of osmotic adjustment and cell wall remodeling represents a fundamental systemic mechanism for maintaining hydraulic integrity in conifers under water deficit.

### 3.11 WGCNA of integrated transcriptomic and metabolomic data

**Figure 8:**
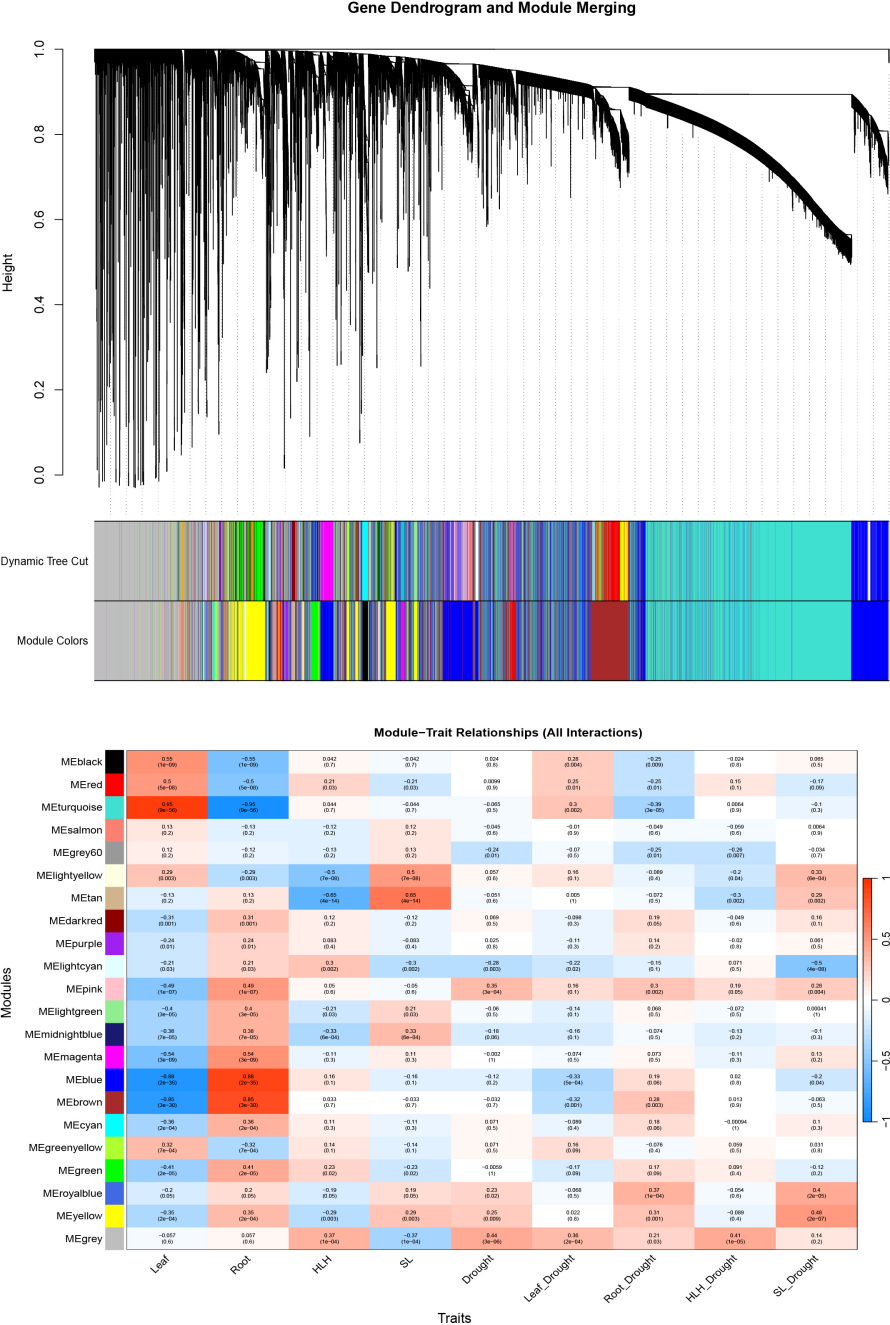
Result of WGCNA. Hierarchical clustering tree showing co-expression modules identified by WGCNA and module sample association relationships.

Weighted Gene Co-expression Network Analysis (WGCNA) was performed on the global transcriptomic and metabolomic datasets, identifying 21 distinct co-expression modules (**Figure 8**; **Supplementary Table 3**). Within the Greenyellow module, Pt5G36740 was identified as a hub gene highly correlated with Jasmonic acid (JA), suggesting a potential link between this gene and JA biosynthesis or metabolism. GO enrichment analysis revealed that the Greenyellow module was predominantly associated with energy metabolism pathways, including ATP synthesis coupled electron transport, oxidative phosphorylation, and NAD metabolic process. Another hub gene within this module, Pt5G36150 (|MM| > 0. 8, kWithin > 8), encodes a ribosomal small subunit protein. Given that protein synthesis is an energy-intensive process, this indicates a close coordination between ribosome-mediated translation and mitochondrial energy supply within this module. Metabolomic evidence further supported this association, with significant enrichment in Glycine, serine and threonine metabolism (*P* = 0. 0001) and Valine, leucine and isoleucine biosynthesis (*P* = 0. 0142). As amino acid catabolism serves as a critical pathway for replenishing the tricarboxylic acid (TCA) cycle to maintain ATP levels under stress, these results confirm the involvement of the Greenyellow module in energy regulation and substrate replenishment during drought.

The Turquoise module was identified as a core regulatory unit for drought response in *P. tabuliformis*, containing the central hub *Pt2G34430* along with key flavonoid regulators, including the activators Pt7G32520 and PtJG27630, and the repressor Pt4G55220. GO enrichment analysis highlighted a highly significant association with photosynthesis (GO:0015979, *P* < 10^–25^), followed by response to water deprivation and response to oxidative stress, further confirming its essential role in stress resistance. Metabolomic analysis corroborated these findings, as metabolites within the Turquoise module were significantly enriched in Flavonoid biosynthesis and Flavone and flavonol biosynthesis. This dual-omics evidence underscores the role of the Turquoise module in regulating flavonoid accumulation. Additionally, the enrichment of Biosynthesis of unsaturated fatty acids (P = 0. 0008) suggests that this module may provide necessary fatty acid substrates for downstream lipid metabolism and the synthesis of signaling molecules such as JA and OPDA.

In the Yellow module, hub genes associated with the accumulation of OPDA precursors (Pt9G09760, Pt1G54060, and Pt9G24870) were identified in conjunction with the osmolyte proline and the cell wall-remodeling laccase gene (Pt5G10920). GO enrichment results showed significant associations with fatty acid oxidation and lipid oxidation, indicating that this module facilitates the supply of precursors for drought-responsive hormonal signaling by producing oxylipins required for OPDA synthesis. The co-expression of proline and laccase genes reflects a coordinated physiological response involving the reduction of cellular osmotic potential through proline accumulation and the reinforcement of cell wall structure via laccase-mediated lignin deposition. Additionally, metabolomic enrichment in isoquinoline alkaloid biosynthesis was observed, highlighting further secondary metabolic involvement in the stress response. In summary, the Yellow module encompasses pathways for hormone precursor supply, osmotic adjustment, cell wall remodeling, and secondary metabolism, representing a central regulatory component for drought adaptation in *P. tabuliformis.*

## 4 Discussion

Previous research on *Pinus tabuliformis* and other conifers has often focused on either transcriptomic-level identification of key gene families (Pervaiz et al., 2021), such as the functional characterization of stress-responsive transcription factors like *PtNAC3* (Han et al., 2023), or metabolomic investigations of physiological responses, such as the comparison of hydraulic architecture responses to warming-drying climates in needles of *Pinus sylvestris* var. mongolica and *P. tabuliformis* by the Shenyang Institute of Ecology (Li et al., 2020). Our study integrates these two approaches, we not only identified differentially expressed genes and metabolites but, more importantly, uncovered potential causal relationships within gene-regulatory metabolic pathways through co-expression network analysis, thereby providing a deeper molecular physiological understanding of drought resistance in *P. tabuliformis*. Specifically, our integrated analysis identified 19 hub genes and 103 hub metabolites that form the core of the drought-responsive network. These hubs, including critical regulators like Pt2G34430 and Pt5G10920, bridge the gap between transcriptomic shifts and metabolic reconfiguration, acting as the primary drivers of the observed physiological adaptations. The root system, as the primary organ for sensing soil drought, has frequently been overlooked in conifer studies due to sampling difficulties (Ding et al., 2025). Even when roots are examined in existing research, the focus is largely confined to physiological aspects like hydraulic structure and carbohydrate reserves. Our research conducted transcriptomic and metabolomic analyses on roots, elucidating their central roles as the first responders to water deficit. Unlike needles that prioritized photosynthesis-related protection, roots exhibited a tissue-specific emphasis on secondary metabolic defense and energy reprogramming. For instance, we found that HLH roots prioritized early activation of Jasmonic Acid-mediated signaling and terpenoid biosynthesis (*PtDXS1*), whereas SL roots demonstrated a significant enrichment in fatty acid β-oxidation. These findings suggest that roots act as a metabolic engine that shifts resources from growth toward energy mobilization and structural reinforcement (via starch and sucrose metabolism) to maintain hydraulic integrity under stress. Research on *Populus tomentosa* has suggested that environmental pressure selects for specific genetic variants, such as the *PtoWRKY68hap1* allele, to aid survival in arid regions (Fang et al., 2023). A similar pattern of adaptation exists in *Pinus tabuliformis* at the physiological level. For instance, northern populations have been found to possess a strong capacity for recovery after drought, whereas southern populations exhibit high resistance but slower recovery rates (Chen et al., 2021). The temporal distribution of DEGs provides evidence for these divergent strategies. In the HLH provenance, the minimal overlap of only 320 shared DEGs between moderate and severe stress stages suggests a high degree of transcriptomic flexibility. This enables the provenance to adjust its physiological state according to the intensity of water deficit. In contrast, the SL provenance exhibited a more continuous molecular response, with 845 genes maintaining differential expression throughout the drought treatment. This pattern indicates a sustained defensive state rather than a rapid adjustment to changing stress levels. This study provides substantial multi-omics evidence for the classical concept of geographic provenance adaptation and offers valuable insights for the precision breeding of drought-resistant tree species.

Different geographic provenances of *P. tabuliformis* have evolved divergent drought response characteristics (Moran et al., 2017; Depardieu et al., 2020). In this study, the HLH provenance, originating from the semi-arid region of Heilihe, Chifeng, Inner Mongolia, is adapted to a precipitation pattern characterized by high uncertainty and short duration summer rainfall, typical of pulsed precipitation (Schwinning and Sala, 2004). To adapt to this environment, the HLH provenance has developed robust growth recovery capabilities. Transcriptomic results indicated that HLH rapidly activated the Jasmonic Acid signaling pathway during early drought to regulate stomatal movement and accumulate defensive compounds (Wasternack and Hause, 2013; Wang et al., 2020). Crucially, upon rehydration, HLH immediately initiated ribosome biogenesis and meristematic phase transition pathways to resume growth. This capacity for rapid recovery is a hallmark of plants from arid regions (Chaves et al., 2009). Conversely, the SL provenance, originating from more southerly regions of the species distribution, adopted a highly dynamic and robust defensive approach, primarily relying on cell wall fortification and the accumulation of flavonoids to prevent water loss. However, this approach required substantial energy reserves. Metabolomic analysis revealed an enrichment of the fatty acid β-oxidation pathway in SL roots, suggesting that these tissues mobilized internal lipids for energy, a phenomenon often associated with depleted energy reserves or severe stress (Xu and Shanklin, 2016). These findings indicate that while the SL provenance attempts to resist drought through defensive structures, the HLH provenance is better adapted to environments characterized by pulsed precipitation and prolonged drought (Yin et al., 2024).

Beyond intraspecific variation, the heterogeneity of response patterns within individual plants reveals a fundamental functional specialization among organs. As the primary site for stress perception, roots prioritized signal transduction and the maintenance of osmotic potential (Takahashi et al., 2020). In this study, this role was characterized by the predominant activation of calcium signaling and MAPK cascades within root tissues, alongside the significant enrichment of starch, sucrose, and amino acid metabolism. Such root-centric osmotic defense, evidenced by the higher accumulation of soluble sugars in roots compared to needles, likely facilitates water uptake by maintaining a favorable water potential gradient. In contrast, the foliar response focused on the inhibition of transpirational loss and the maintenance of cellular homeostasis. Leaves achieved a physical barrier to water loss by modulating stomatal aperture and reinforcing the thickness of the cuticle (Xue et al., 2017), leaves exhibited a marked induction of flavonoid and terpenoid biosynthetic pathways, particularly during prolonged stress. This metabolic shift in the needles, supported by the upregulation of antioxidant-related pathways, likely serves as a protective mechanism to scavenge reactive oxygen species generated by photo-oxidation. The synchronization between root-mediated water supply and leaf-mediated conservation of internal water serves as the core coordination required to preserve whole-plant hydraulic integrity during the early stages of drought (Brodribb et al., 2020).

Transcriptomic analysis revealed that pathways associated with biotic stress resistance, specifically defense response to insect (GO:0002213) and defense response to Gram-negative bacterium (GO:0050829), were significantly enriched in *P. tabuliformis* leaves during the mild drought stage. This transcriptional activation occurred in a controlled experimental environment devoid of pathogens or herbivores, reflecting an adaptive evolutionary strategy characteristic of evergreen conifers (Coley, Bryant, and Chapin 1985). In natural ecosystems, climate change drivers often exert synergistic effects on forest health, where drought stress can compromise host tree resistance and render them more susceptible to secondary pest outbreaks (Jactel, Koricheva, and Castagneyrol 2019). Consequently, the observed upregulation of biotic defense pathways likely represents a pre-emptive compensatory mechanism. By activating these defenses before severe physiological damage occurs, *P. tabuliformis* may counteract the anticipated risk of herbivory that frequently accompanies drought events (Neilson et al., 2013; Jactel et al., 2012). The data presented here suggest that *P. tabuliformis* has evolved a mechanism to activate biotic defense systems in response to abiotic water deficit signals (Fujita et al., 2006). Following the transcriptional upregulation of defense genes during mild drought, a substantial accumulation of secondary metabolites was observed under severe drought conditions, including flavonoids synthesized via flavonol synthase activity (GO:0045431) and terpenoids synthesized via dimethylallyltranstransferase activity (GO:0004161). Flavonoids function as antioxidants to mitigate drought-induced oxidative stress while simultaneously acting as feeding deterrents against insects (Treutter, 2006). Similarly, terpenoids constitute the chemical basis of oleoresin and provide a toxic barrier against pests (Keeling and Bohlmann, 2006). The proactive synthesis of these chemical defenses during the early and intermediate phases of drought minimizes the physiological lag time required to respond to herbivore attacks when seedlings are in a weakened physiological state. This coordinated regulation of drought tolerance and biotic stress resistance represents a critical survival strategy for maintaining population stability in fluctuating environments (Mumm and Hilker, 2006).

Weighted gene co-expression network analysis (WGCNA) revealed potential molecular regulatory associations in *Pinus tabuliformis* under drought stress. The correlation between the hub gene *Laccase* (Pt5G10920) and proline accumulation suggests a possible link between cell wall fortification and osmotic adjustment (Zhao et al., 2013; Cesarino, 2019). Laccases are involved in lignin polymerization within the apoplast to enhance structural strength, while proline functions to maintain intracellular turgor (Szabados and Savouré, 2010). The synchronization of these processes may represent a candidate mechanism for maintaining structural integrity and preventing mechanical collapse under the negative hydraulic pressure induced by drought. Furthermore, the data point toward a potential molecular link for stomatal regulation. The stress-responsive gene Pt5G36740 (a *LURP* homolog) was significantly positively correlated with the vacuolar malate transporter (Pt0G36800). Given that malate flux across the tonoplast is essential for the regulation of guard cell movement (Eisenach and De Angeli, 2017), this association provides a plausible molecular explanation for the translation of internal signals into the physiological arrest of transpiration. Additionally, a potential negative regulator in the flavonoid pathway (Pt4G55220) was identified, which may assist in maintaining energy homeostasis by preventing excessive resource expenditure during prolonged stress (Xu et al., 2015) These identified hub genes and their associated networks provide valuable candidates for the genetic improvement of drought resistance in conifers.

Although this integrated analysis provided several insights, certain limitations must be acknowledged. This study was conducted under controlled experimental conditions, which may not fully replicate the complex stress environments found in natural forest ecosystems. Furthermore, while multiple hub genes were identified through the co-expression network, their definitive functions remain hypothetical and requires functional validation using transgenic or gene-editing approaches. Future research should prioritize the functional characterization of these candidate genes to confirm their specific contributions to drought adaptation. Additionally, expanding this research to include a broader diversity of provenances and long-term field observations is essential to validate the ecological significance of the mechanisms identified in this study.

## 5 Conclusion

In this study, changes in antioxidant enzyme activities and leaf relative water content of Pinus tabuliformis were investigated. Integrated transcriptomic and metabolomic analyses were performed to characterize gene expression patterns and metabolite content variations during drought stress and rehydration. Drought resistance mechanisms were compared between two *P. tabuliformis* provenances (HLH and SL) from distinct ecological environments, as well as between roots and needles. Time-series transcriptomic analysis identified several potential key genes, including Pt2G21940, PtQG34150, Pt1G23820, and Pt1G23830. Additionally, gene regulatory networks associated with proline, jasmonic acid, and flavonoid metabolites were analyzed. These key genes and potential networks serve as focal points for future drought tolerance research in *P. tabuliformis*, providing a theoretical basis for understanding the molecular mechanisms underlying drought resistance in conifers.

## Data Availability

The raw data supporting the conclusions of this article will be made available by the authors, without undue reservation.
